# Clinico-pathological discrepancies in the diagnosis of causes of death in adults in Mozambique: A retrospective observational study

**DOI:** 10.1371/journal.pone.0220657

**Published:** 2019-09-06

**Authors:** Jaume Ordi, Paola Castillo, Alberto L. Garcia-Basteiro, Cinta Moraleda, Fabiola Fernandes, Llorenç Quintó, Juan Carlos Hurtado, Emili Letang, Lucilia Lovane, Dercio Jordao, Mireia Navarro, Rosa Bene, Tacilta Nhampossa, Mamudo R. Ismail, Cesaltina Lorenzoni, Assucena Guisseve, Natalia Rakislova, Rosauro Varo, Lorena Marimon, Ariadna Sanz, Anelsio Cossa, Inacio Mandomando, Maria Maixenchs, Khátia Munguambe, Jordi Vila, Eusebio Macete, Pedro L. Alonso, Quique Bassat, Miguel J. Martínez, Carla Carrilho, Clara Menéndez

**Affiliations:** 1 Department of Pathology, Hospital Clínic of Barcelona, University of Barcelona, Barcelona, Spain; 2 ISGlobal, Hospital Clínic - Universitat de Barcelona, Barcelona, Spain; 3 Centro de Investigação em Saúde de Manhiça (CISM), Maputo, Mozambique; 4 Amsterdam Institute for Global Health and Development (AIGHD), Academic Medical Center, Amsterdam, The Netherlands; 5 Department of Pathology, Maputo Central Hospital, Maputo, Mozambique; 6 Faculty of Medicine, Eduardo Mondlane University, Maputo, Mozambique; 7 Department of Microbiology, Hospital Clinic of Barcelona, University of Barcelona, Barcelona, Spain; 8 Service of Infectious Diseases, Hospital del Mar, Hospital del Mar Research Institute (IMIM), Barcelona, Spain; 9 Department of Medicine, Maputo Central Hospital, Maputo, Mozambique; 10 ICREA, Catalan Institution for Research and Advanced Studies, Barcelona, Spain; 11 Pediatric Infectious Diseases Unit, Pediatrics Department, Hospital Sant Joan de Déu, University of Barcelona, Barcelona, Spain; 12 Consorcio de Investigación Biomédica en Red de Epidemiología y Salud, Madrid, Spain; Fundacao Oswaldo Cruz, BRAZIL

## Abstract

**Background:**

Clinico-pathological discrepancies are more frequent in settings in which limited diagnostic techniques are available, but there is little information on their actual impact.

**Aim:**

We assessed the accuracy of the clinical diagnoses in a tertiary referral hospital in sub-Saharan Africa by comparison with post-mortem findings. We also identified potential risk factors for misdiagnoses.

**Methods:**

One hundred and twelve complete autopsy procedures were performed at the Maputo Central Hospital (Mozambique), from November 2013 to March 2015. We reviewed the clinical records. Major clinico-pathological discrepancies were assessed using a modified version of the Goldman and Battle classification.

**Results:**

Major diagnostic discrepancies were detected in 65/112 cases (58%) and were particularly frequent in infection-related deaths (56/80 [70%] major discrepancies). The sensitivity of the clinical diagnosis for toxoplasmosis was 0% (95% CI: 0–37), 18% (95% CI: 2–52) for invasive fungal infections, 25% (95% CI: 5–57) for bacterial sepsis, 34% (95% CI: 16–57), for tuberculosis, and 46% (95% CI: 19–75) for bacterial pneumonia. Major discrepancies were more frequent in HIV-positive than in HIV-negative patients (48/73 [66%] vs. 17/39 [44%]; p = 0.0236).

**Conclusions:**

Major clinico-pathological discrepancies are still frequent in resource constrained settings. Increasing the level of suspicion for infectious diseases and expanding the availability of diagnostic tests could significantly improve the recognition of common life-threatening infections, and thereby reduce the mortality associated with these diseases. The high frequency of clinico-pathological discrepancies questions the validity of mortality reports based on clinical data or verbal autopsy.

## Introduction

Healthcare systems worldwide face the challenge of improving the quality of care they deliver in order to improve health outcomes. Complete autopsy is the gold standard methodology for cause of death investigation and is the most informative tool for retrospective quality assessment of clinical diagnoses [1,2]. Previous studies comparing clinical diagnoses and autopsy findings revealed major discrepancies in approximately 10–40% of the deceased patients undergoing post-mortem examination [3–5]. Thus, complete diagnostic autopsy is an important tool for quality control of clinical practice [6,7].

However, autopsy rates have been declining over the past decades [3–5,8,9]. The reasons for this decline have been discussed previously in literature [3,4,6] and include, among others, introduction of modern diagnostic tools in clinical practice. However, it is widely accepted that clinico-pathological discrepancies still occur even in the era of high-tech medicine [7,8,10], being much more frequent in low and middle income countries (LMIC), in which the availability of diagnostic techniques is limited [9]. Therefore, autopsies still have a crucial role in quality control of the clinical diagnosis and overall patient management. Learning from individual and system-related diagnostic errors can contribute to improving the quality of care [4].

In this study, we assessed the concordance between clinical and autopsy diagnoses by determining the major and minor discrepancy rates in a total of 112 consecutive autopsies performed in adults, excluding maternal deaths, conducted at the Maputo Central Hospital (MCH) in Mozambique. We analysed the influence of several factors on the frequency of major discrepancies, including diagnostic category, HIV infection and length of time from admission to death. We also evaluated the association between clinical signs and symptoms and frequency of errors. These results could contribute to identifying future patients at a higher risk of misdiagnosis and may help to define strategies to implement diagnostic tests to confirm or exclude frequent diseases causing death.

## Material and methods

### Study area and design

This was an observational study performed from November 2013 to March 2015 at the Department of Pathology in collaboration with the Department of Internal Medicine of the Maputo Central Hospital, a 1500-bed public tertiary health care centre in Maputo city, the capital of Mozambique. The study was part of the validation of minimally invasive autopsy (MIA) against the gold standard complete autopsy [11–14]. The inclusion criteria were: 1) a complete autopsy requested by the clinician as part of the medical evaluation of the patient; 2) verbal informed consent to perform the autopsy given by the relatives; and 3) age older than 15 years. Traumatic and maternal deaths were excluded from this analysis. From November 2013 to March 2015, two autopsies were prospectively included per day. In order to select these two cases per day among the daily autopsy requests received at the department (between 5 and 12 per day) without introducing selection biases, the two patients with death recorded closest to before the time of 8.00 a.m. were included in the study.

### Autopsy procedures and laboratory analyses

A detailed description of the pathological and microbiological methodology of the complete autopsies has been reported previously [11–14]. Briefly, a dissection was performed with macroscopic evaluation of all the organs following a standardized macroscopic protocol [15]. Samples of blood, cerebrospinal fluid (CSF), liver, lungs, bone marrow, central nervous system (CNS), heart, kidney, spleen and any grossly identified lesion were obtained. Samples were obtained from all the organs for microbiological and histological analyses.

Macroscopic and microscopic autopsy data were evaluated by a team of two pathologists. For histologic examination, all samples were routinely stained with haematoxylin and eosin. Complementary histochemical and/or immunohistochemical stains were also performed if required for the diagnosis. Two microbiologists analysed the microbiological results according to methodology described elsewhere [13]. Briefly, universal screening was performed in all the cases, including the detection of *Plasmodium falciparum* by PCR, detection of antibodies against human immunodeficiency virus (HIV)-1/2, as well as cultures for bacteria and fungi using blood and CSF samples. Determination of viral load was also performed in samples tested positive for antibodies against HIV. Additional microbiological screening tests were included in all HIV-positive cases, including real time PCR in CSF, CNS and lung for *Toxoplasma gondii*, *Mycobacterium tuberculosis* and *Cryptococcus* spp. and in lung samples for *Pneumocystis jirovecii*. Other microorganisms were also tested according to the pathological findings observed in the tissues obtained during the MIA procedure.

### Determination of the cause of death

A team composed of a pathologist, a microbiologist, and a clinician with expertise in infectious diseases and epidemiology evaluated all the data of the autopsy (macroscopic findings, histological reports and microbiological results) in combination with clinical data, and assigned the final diagnosis of cause of death (gold standard).

All morbid conditions directly leading to death, underlying conditions (if present), and any other conditions that could contribute to death were codified according to the International Classification of Diseases, Tenth Revision (ICD-10). Diagnoses were categorised into the following diagnostic groups: tuberculosis, invasive fungal infections, toxoplasmosis, bacterial sepsis, disseminated viral infections, bacterial pneumonias, viral pneumonias, pericarditis, bacterial meningitis, viral meningoencephalitis, gastrointestinal infections, malignant tumours and other non-infectious diseases (cardiovascular, kidney, gastrointestinal and pulmonary diseases). The main final diagnosis was used to define the disease category.

### Review of the clinical charts

Clinical information from all the patients recruited was retrospectively abstracted using a standardised questionnaire (S1 Form). The same investigator (Q.B.) performed the clinical data abstraction of all cases after thorough revision of the entire medical record. The data collection included, among others, retrieving demographic data, past medical history, as well as information about the inpatient admission process and the clinical information of the disease during hospitalisation, including signs and symptoms, physical examination, laboratory results, imaging results and treatment received. Up to five clinical diagnoses registered in the medical record were selected. We assumed that first diagnosis listed in the clinical record was the principal diagnosis, and the remaining diagnoses were classified as secondary. All treatments received by the patients during admission were also evaluated.

### Assessment of discrepancies between clinical and autopsy diagnoses

Clinico-pathological discrepancies were classified according to the criteria of Goldman modified by Battle, and as non-classifiable cases [16–18]. Major discrepancies were directly related to death and were grouped as class I or class II errors. As described previously [19], class I error had a direct impact on the survival of the patient t (e.g., pulmonary infarction treated as pneumonia), while class II error, if recognized, would not have altered the survival of the patient (e.g., missed pneumonia in a patient with disseminated malignant neoplasm). Class III (non-diagnosed diseases that would have eventually affected the prognosis) and class IV discrepancies involved minor diagnoses, unrelated to cause of death [19]. Concordant diagnoses were classified as class V and non-classifiable cases were included in class VI (e.g., autopsy with no-conclusive diagnosis).

Each case was assessed blindly by two investigators (C.Mo., A.L.G.B.) and the two interpretations were compared. Discrepant cases were evaluated by a third rater (C.Me.). Each rater was provided with an excel file containing the following information: a) autopsy final diagnosis, antecedent causes, and other significant conditions; b) clinical diagnoses extracted from the medical record including up to five diagnoses. Assessment of the clinical errors was made unidirectionally, that is, looking at whether the autopsy diagnosis was identified among the clinical diagnoses, regardless of whether there were other clinical diagnoses not confirmed by the autopsy. A case was classified as a major discrepancy only when there was no coincidence between any of the five clinical diagnoses listed by the clinicians and the final cause of death identified at the autopsy. In each case, only the worst diagnostic error was considered. The etiological agent was considered to assess a clinic-pathological discrepancy for specific infectious diseases including tuberculosis, toxoplasmosis, and invasive fungal diseases. Once the assessment of the clinic-pathological discrepancy had been completed, the treatments received by the patients were evaluated in order to verify the coherence with the clinical diagnoses.

### Definitions and statistical methods

False-negative cases contained class I and II discrepancies in which the autopsy diagnosis was in the assessed diagnostic category but the clinical diagnosis was in another category. False-positive diagnoses were cases with major discrepancies (class I and II), in which the clinical diagnosis was in the assessed diagnostic category but not the autopsy diagnosis. The concordance between raters was assessed by the Kappa statistic, which adjusts for chance agreement, and interpreted as suggested by Landis and Koch [20]. Proportions were compared by chi-square test, and logistic regression with penalised likelihood was used to evaluate factors associated with major clinical errors [21,22].The sensitivity, specificity, positive (PPV) and negative predictive values (NPV) for each diagnosis were calculated. Data were analysed with the STATA program (Version 15, StataCorp 2017, College Station, TX, USA).

## Results

The study included 112 autopsies performed in adults (57 males and 55 females, median age 37 years, range 15–76). Seventy-three out of the 112 patients (65%) tested positive for antibodies against HIV in the blood obtained at complete autopsy. The final cause of death was attributed by the autopsy to infectious disease in 80/112 (71%) cases, followed by malignant tumours in 16/112 (14%) and other non-infectious disease in 16/112 (14%) cases.

In 87 out of the 112 cases, the two raters attributed the same type of error (78% agreement). The kappa value between the two evaluations was 0.6520 (95% CI 0·5423–0.7616, p<0.0001, substantial agreement). A discordant evaluation was observed in 25 cases (22%), thus requiring a third evaluation.

Overall, diagnostic discrepancy was observed in 71/112 (63%) of the cases. A major diagnostic discrepancy was detected in 65 (58%) deaths, 63 (56%) of which were classified as class I, and two (2%) as class II. A minor diagnostic discrepancy (class III or IV) was identified in six cases (5%) and included one class III, and five class IV errors. In 41 deaths (37%), there was complete agreement between the clinical and the autopsy diagnoses (class V). No time trends concerning the clinical-pathological discrepancies were identified throughout the years (25/45 [56%] of major errors in 2013 vs. 40/67 [60%] in 2014; p = 0.6629).

Table 1 shows the causes of death detected in the autopsy, the number of clinical diagnoses for each cause of death suspected pre-mortem by the clinician, the number and percentage of false negative (major diagnostic errors) and false positive diagnoses, as well as the sensitivity, specificity, PPV, and NPV of the clinical diagnosis for each diagnostic category.

**Table 1:** Causes of death determined by the autopsy and the clinical diagnosis, absolute numbers and percentages of false negative and false positive clinical diagnoses and sensitivity, specificity, PPV, and NPV of the clinical diagnosis for each diagnostic category.

	Autopsy diagnosis	Clinical diagnosis	False negative diagnosis		False positive diagnosis		Sensitivity		Specificity		Positive predictive value		Negative predictive value	
	n	n	n	%	n	%	%	95%CI	%	95%CI	%	95%CI	%	95%CI
Tuberculosis	23	19	15	65	11	12	34	16–57	88	79–94	42	20–66	84	75–91
Invasive fungal infections	11	7	9	82	5	5	18	2–52	95	89–98	29	4–71	91	84–96
Toxoplasmosis	8	2	8	100	2	2	0	0–37	98	93–100	0	0–84	93	86–97
Bacterial sepsis	12	5	9	75	2	2	25	5–57	98	93–100	60	15–95	92	85–96
Disseminated herpesvirus	1	1	0	0	0	0	100	2–100	100	97–100	100	2–100	100	97–100
Bacterial pneumonia	13	21	7	54	15	15	46	19–75	85	76–91	29	11–52	92	85–97
Viral pneumonia	2	0	2	100	0	0	0	0–84	100	97–100	-	-	98	94–100
Pericarditis	1	1	0	0	0	0	100	2–100	100	97–100	100	2–100	100	97–100
Bacterial meningitis	4	18	3	75	17	16	25	1–81	84	76–91	6	0–27	97	91–99
Viral meningoencephalitis	3	4	2	67	3	3	33	1–91	97	92–99	25	1–81	98	93–100
Gastrointestinal infection	2	6	1	50	5	5	50	1–99	95	90–98	17	0–64	99	95–100
Malignant tumours	16	24	5	31	13	14	69	41–89	86	78–93	46	26–67	94	87–98
Cardiovascular disease	11	19	1	9	9	9	91	59–100	91	84–96	53	29–76	99	94–100
Gastrointestinal diseases	2	3	1	50	2	2	50	1–99	98	94–100	33	1–91	99	95–100
Kidney disease	1	6	1	100	6	5	0	0–97	95	89–98	0	0–46	99	95–100
Pulmonary disease	2	1	1	50	0	0	50	1–99	100	97–100	100	2–100	99	95–100

CI: Confidence interval

A major discrepancy was observed in 56/80 (70%) of the deaths of infectious origin. The diagnosis of tuberculosis was not clinically suspected in 15/23 cases (65% major clinical discrepancies). A major clinical error was observed in 9/16 (56%) of miliary tuberculosis, 4/5 (80%) of pulmonary tuberculosis and 2/2 (100%) of tuberculous meningitis.

A diagnosis of invasive fungal infection was clinically unsuspected in 9/11 cases (82% major clinical discrepancies). A major clinical error was identified in 5/7 cryptococcosis, 2/2 pulmonary pneumocystosis, 1/1 mucormycosis and 1/1 disseminated candida infection). None of the eight cases of toxoplasmosis confirmed in the autopsy was clinically suspected (100% major clinical discrepancies).

The diagnosis of bacterial sepsis was not clinically suspected in 9/12 cases (75% major clinical discrepancies). Bacterial pneumonia was not clinically suspected in 7/13 cases (54% major discrepancies). Bacterial infections of the CNS were not clinically suspected in 4/7 cases (57% major clinical discrepancies).

The diagnosis of malignant tumour was missed in 5/16 cases (31% major discrepancies; 0/5 liver cell carcinoma, 0/3 carcinoma of the uterine cervix, 1/3 malignant lymphomas, 1/2 Kaposi’s sarcoma, 1/1 myeloid leukaemia, 1/1 brain tumour, 1/1 undifferentiated malignant tumour of unknown primary).

The diagnosis of other non-infectious disease was not suspected as a cause of death in 4/16 cases (25% major discrepancies; 1/11 cardiovascular disease, 2/3 renal and gastrointestinal diseases and 1/2 pulmonary diseases).

“Eighty-six of the patients died at the internal medicine wards, 15 at the emergency room and 11 at the intensive care unit. No differences were observed in percentage of major errors between deceases occurring at the different units (49/86 [57%], 9/15 [60%] and 7/11 [64%], respectively, p = 0.9465).”

S1 Table shows the age, the sex, the HIV status as reported in the clinical records, the clinical diagnoses (up to five) registered in the clinical records, the autopsy diagnosis, the HIV status at autopsy, and the final assessment of the clinico-pathological discrepancy of each particular patient. Meningoencephalitis was the most common false positive diagnosis. Although some of the diseases established as the autopsy diagnosis could cause meningoencephalitic symptoms (e.g. tuberculosis, cryptococcosis, etc.), none of these patients had received adequate treatment for the disease causing death.

HIV infection was clinically known in 62 out of the 73 patients (85%) in whom HIV antibodies were identified in the autopsy, whereas HIV had not been documented in 11 patients. Six of the 11 cases with no HIV test performed pre-mortem had died within 24 hours of admission. According to the clinical records, only 48/73 HIV-positive patients (66%) had received antiretroviral therapy prior to admission. Table 2 shows the distribution of the clinico-pathological discrepancies and the main cause of death according the HIV status. The percentage of major errors in HIV-positive patients was higher than in HIV-negative patients.

**Table 2:** Distribution of the clinico-pathological discrepancies and the main cause of death according the HIV status.

	HIV status		
	Negative	Positive	
Clinico-pathological error	N %	N %	p-value
Class I	16 (41%)	47(64%)	0.0059 ^2^
Class II	1 (3%)	1 (1%)	
Class III	1 (3%)	0 (0%)	
Class IV	0 (0%)	5 (7%)	
Class V	21 (54%)	20 (27%)	
**Type of error**			
Major	17 (44%)	48 (48%)	0.0236 ^3^
None/Minor	22 (56%)	25 (34%)	
**Diagnostic group**			
Infectious disease	21 (26%)	59 (74%)	0.0009 ^3^
Malignant neoplasm	6 (38%)	10 (62%)	
Other disease	12 (75%)	4 (25%)	

^2^: Fisher’s exact test

^3^: Chi-square test

Information on the latest CD4 T-cell counts was available in 21/73 HIV positive subjects. No differences in percentage of major errors were observed between patients with <200 and >200 CD4 cells/mm3 (6/8, 75% vs. 12/13, 93%; p = 0.5308). No differences were observed wither in relation to viral load (5/7, 71% in patients with >100,000 copies, 28/46, 61% in patients with 15–100,000 copies, 14/20, 70% in patients with positive HIV antibodies and no quantifiable viral copies; p = 0.7542). Information on prophylactic cotrimoxazol was available in 55/73 HIV positive subjects. No differences were observed between patients receiving or not prophylactic cotrimoxazol (17/29, 59% vs. 18/26 69%; p = 0.5752).

Overall, 38 patients (34%) died within 24 hours of admission, 19 (17%) between 24–48 hours and 53 (47%) more than 48 hours after admission. No data on time between admission and death was available in two cases (2%). No statistically significant association was identified between time from admission to death and percentage of major discrepancies (24/38, 63% major clinical errors in patients who died within 24 hours of hospitalisation, and 40/72; 55% in patients who died after 24 hours of hospitalisation, p = 0.4421; 34/57, 60%, major clinical errors in patients who died within 48 hours vs. 30/53, 57%, in patients who died after 48 hours of hospitalisation, p = 0.7463).

The most relevant associations between demographic data, clinical symptoms at admission and biochemical and imaging results, and type of clinic-pathological discrepancies are shown in Table 3. The table includes all statistically significant associations, as well as some relevant demographic data. The crude model, as well as the model adjusted for HIV status is presented. In the crude analysis, major discrepancies were most frequent in patients who presented headache and had been under antiretroviral treatment. No association was identified between other demographic data (urban or rural origin, ethnic group, smoking habit, and alcoholism), symptoms and signs at admission (cough, dyspnea, diarrhea, vomiting, convulsions, abdominal pain, oliguria, dysuria, haematuria, diabetes, hypertension, treatments received before admission, weight, nutritional status, axillar temperature, dehydration, oedema, adenopathy, pallor, jaundice, hepatomegaly, splenomegaly, petechial rash, exanthema, coma), biochemical and imaging results (anaemia, leukocytes, urine, liver and renal analytical data) and treatments received during hospitalisation. When adjusting for HIV status only previous antiretroviral treatment was statistically significant.

**Table 3:** Relevant associations between demographic data, clinical symptoms at admission and biochemical and imaging results, and type of clinico-pathological discrepancies. The crude model, as well as the model adjusted for HIV status is presented.

	Type of error		Crude model ^1^		Adjusted model ^1^	
	None/Minor (N = 47)	Major (N = 65)	Odds Ratio (95%CI)	p-value	Odds Ratio (95%CI)	p-value
**Sex** ^2^						
Male	24 (51%)	34 (52%)	1	-	1	-
Female	23 (49%)	31 (48%)	0.9 (0.4–2.0)	0.8967	0.9 (0.3–2.9)	0.8529
**Age at death (years)** ^3^	41.4 (13.7)	38.0 (13.0)	1.0 (0.9–1.0)	0.1972	1.0 (0.9–1.1)	0.9949
**Elapsed time between admission and death (hours)** ^3^	106.7 (125.4)	95.2 (122.3)	1.0 (1.0–1.0) ^5^	0.6203	1.0 (1.0–1.0)	0.7869
**Fever prior to admission** ^2^	16 (34%)	33 (51%)	2.0 (0.9–4.2)	0.0835	0.7 (0.2–2.5)	0.6155
**Headache** ^2^	6 (13%)	23 (35%)	3.5 (1.3–9.3)	0.0107	4.5 (0.7–27.8)	0.1058
**Antiretroviral treatment** ^2^	14/29 (48%)	34/41 (83%)	4.9 (1.7–14.3)	0.0034	5.6 (1.5–2.5)	0.0114
**CoD group** ^2^						
Infectious diseases	24 (51%)	56 (86%)	1	0.0007	1	0.0620
Malignant neoplasms	11 (23%)	5 (8%)	0.2 (0.1–0.6)		0.2 (0.0–0.9)	
Other diseases	12 (26%)	4 (6%)	0.2 (0.1–0.5)		0.2 (0.0–1.7)	
**HIV-status** ^2^	25 (53%)	48 (74%)	2.4 (1.1–5.4)		2.2 (0.4–12.8)	

^1^: Penalized-Logistic regression

^2^: absolute number (percentage)

^3^: Arithmetic Mean (Standard Deviation)

## Discussion

To our knowledge, this is the first study conducted in sub-Saharan Africa focused on clinico-pathological discrepancies based on complete diagnostic autopsy and including extensive serological and microbiological evaluation comprising HIV testing, as well as classical cultures and molecular tests for bacteria, fungi, viruses, and parasites. There was a high frequency of clinico-pathological discrepancies in this series of adult autopsies carried out in a tertiary-referral hospital in Mozambique, with most being major errors. Importantly, in most patients a change in clinical management could have significantly modified the prognosis (class I errors). The proportion of discrepancies was higher than the rate reported in most studies [18,23–25], but similar to the percentage observed in our previous study on maternal deaths conducted in 2009 in the same institution (Maputo Central Hospital) and also based on complete autopsy [19]. Clinical errors were more frequent in patients with infectious diseases than in patients with tumours or other diseases. A high rate of false negative diagnoses was found for infectious diseases (100% for toxoplasmosis, 82% for fungal infections, 75% for bacterial sepsis and bacterial meningitis, 65% for tuberculosis, and 54% for bacterial pneumonia). Interestingly, the only clinical symptom associated with an increased frequency of major errors was headache, probably related to the high rate of false negative diagnoses in patients with infections causing meningoencephalitis such as toxoplasmosis and cryptococcosis. No association was found between the time from admission to death and the percentage of major discrepancies.

Previous studies have also shown higher rates of diagnostic errors for infectious diseases than other conditions [18,23–25]. False negative results can be due to underestimation of the prevalence of the infectious diseases, inadequate synthesis in the diagnostic process, and the poor sensitivity of the diagnostic tests available [26–28]. The results of our study suggest that adequate knowledge of the prevalence of infectious diseases along with an increased level of suspicion for most frequent infections and the implementation of accurate, low-cost, diagnostic tools may lead to decline of major and minor diagnostic errors [19,26,29] and may have an impact on decreasing mortality rates.

Interestingly, the percentage of major discrepancies was higher in HIV-positive than in HIV-negative patients. Diagnostic discrepancies rates in HIV positive patients are high in other sub-Saharan countries [30]. In addition, the high frequency of multiple microbiological agents in HIV-positive patients makes clinical diagnosis difficult and contributes to misdiagnosis or misinterpretation of complementary diagnostic methods [30]. In 85% of the HIV-positive patients, the infection had been adequately identified during life; however, only 66% of these patients were on antiretroviral therapy. Limited access to necessary diagnostic tests in support of HIV/AIDS and associated co-infections, such as CD4 cell counts, viral load, tuberculosis microscopy or molecular diagnosis, drug susceptibility testing, and cryptococcal antigen testing among others, negatively contributes to the adequate clinical diagnosis, and consequently delays initiation of drug therapy in LMIC, particularly in sub-Saharan Africa [31,32]. These features have a critical impact on life expectancy. Laboratory infrastructure and capacity are sub-optimal or do not exist at all in rural settings of many LMIC, which results in a very limited diagnostic capacity. As a consequence, treatment is commonly administered in the absence of diagnostic testing, which, in addition to the possible inadequacy of the drug, potentially accelerates the incidence of drug-related toxicity and the onset of drug resistance. Barriers for the expansion of necessary diagnostic testing of major infectious diseases include a severe deficit of qualified laboratory personnel, inadequate training and insufficient educational programmes for specific diagnostic tests, inadequate standards and accreditation systems for different type of laboratories and a lack of quality control programmes. All these limitations interfere with the quality of the tests. The cost of diagnostic equipment and consumables are also challenging in most LMIC [33]. Financial constraints hamper improvements in laboratory services, maintenance of equipment, and the procurement of laboratory supplies.

The significant reduction of diagnostic errors reported in high-income countries in the last decades has mainly been attributed to the improvement of clinical skills and to the impact of new diagnostic procedures [26]. Autopsy has the dual role of a method whereby diagnostic errors can be detected and a source of knowledge to be applied to future cases, and has influenced learning and provided additional data on local epidemiology of diseases. The almost complete absence of studies based on autopsy data and focused on diagnostic errors is a severe handicap for medical practice in sub-Saharan Africa. The increase of autopsy studies could help to reduce mortality by providing information critical to improve diagnostic accuracy and, therefore, clinical management.

Our study has some limitations. First, because our study was conducted in a large national reference and university tertiary hospital, the findings observed cannot be extrapolated to other healthcare facilities since a lower frequency of clinico-pathological discrepancies has been reported in tertiary hospitals [18]. Thus, in primary or small health facilities the number of diagnostic errors could be higher. On the other hand, complicated or severely ill patients are often referred to large hospitals resulting in a high number of cases of increased diagnostic difficulty. Secondly, similarly to what has been reported in other studies [26], we found a disagreement rate of 22% in class assignment, which reflects the difficulties in classifying the discrepancy classes in some cases, which could be a limitation of the discrepancy classification.

Importantly, major clinico-pathological discrepancies may have important implications for research. It is well known that the main source of information on the causes of death in LMIC is verbal autopsy (method in which the cause of death is determined by analysis of interviews to relatives or caretakers of the deceased) [34]. Obviously, verbal autopsy has been questioned because it is subject to a relatively high degree of misclassification error [35,36]. Our data indicate that medical records, and, probably also verbal autopsy diagnoses contain important a significant proportion of inaccuracies [37]. Interestingly, the studies based on these methodologies tend to underreport infectious diseases [38], especially in sub-Saharan Africa, vital statistics, clinical registries, randomised trials, mortality reports and health policy decision, might be based on incorrect causes of death, as autopsy is rarely the source of information.

In conclusion, major clinico-pathological discrepancies are still frequent in resource constrained settings including tertiary referral institutions in sub-Saharan Africa. Increasing the level of clinical suspicion for most frequent infectious diseases and introduction of simple diagnostic tests could significantly improve the recognition of common and life-threatening infections, and thereby reduce the associated mortality. The high frequency of clinico-pathological discrepancies questions the validity of mortality reports based on clinical data or verbal autopsy.

## Supporting information

S1 Table Age, sex, HIV status in the clinical records, clinical diagnoses (up to five) registered in the clinical records, autopsy diagnosis, HIV status at autopsy and final assessment of the clinico-pathological discrepancy of each particular patient. (DOCX)

S1 Form Standardized questionnaire used to collect clinical data of each particular patient. (PDF)

## References

[1] Shojania KG, Burton EC, McDonald KM, Goldman L. Changes in rates of autopsy-detected diagnostic errors over time: a systematic review. JAMA 2003;289:2849–56. 10.1001/jama.289.21.2849 12783916

[2] Shojania KG, Burton EC. The vanishing nonforensic autopsy. NEnglJMed 2008;358:873–5. 10.1056/NEJMp0707996 18305264

[3] Hinduja A, Gupta H, Dye D. Autopsy proven causes of in hospital mortality in acute stroke. J Forensic Leg Med 2013;20:1014–7. 10.1016/j.jflm.2013.09.020 24237810

[4] Kuijpers CC, Fronczek J, van de Goot FR, Niessen HW, van Diest PJ, Jiwa M. The value of autopsies in the era of high-tech medicine: discrepant findings persist. JClinPathol 2014;67:512–9. 10.1136/jclinpath-2013-202122 24596140

[5] Wittschieber D, Klauschen F, Kimmritz A-C, von Winterfeld M, Kamphues C, Scholman H-J, et al Who is at risk for diagnostic discrepancies? Comparison of pre- and postmortal diagnoses in 1800 patients of 3 medical decades in East and West Berlin. PLoS One 2012;7:e37460 10.1371/journal.pone.0037460 22629399 PMC3358345

[6] Bansal MG, Punia RS, Sachdev A. Clinical and Needle Autopsy Correlation Evaluation in a Tertiary Care Teaching Hospital: A Prospective Study of 50 Cases From the Emergency Department. Am J Forensic Med Pathol 2012;33:194–6. 10.1097/PAF.0b013e31823d295e 22543521

[7] Liu D, Gan R, Zhang W, Wang W, Saiyin H, Zeng W, et al Autopsy interrogation of emergency medicine dispute cases: how often are clinical diagnoses incorrect? J Clin Pathol 2018;71:67–71. 10.1136/jclinpath-2017-204484 28735302

[8] Murken DR, Ding M, Branstetter BF, Nichols L. Autopsy as a quality control measure for radiology, and vice versa. AJR Am J Roentgenol 2012;199:394–401. 10.2214/AJR.11.8386 22826402

[9] Schwanda-Burger S, Moch H, Muntwyler J, Salomon F. Diagnostic errors in the new millennium: a follow-up autopsy study. Mod Pathol 2012;25:777–83. 10.1038/modpathol.2011.199 22362052

[10] Roberts ISD, Benamore RE, Benbow EW, Lee SH, Harris JN, Jackson A, et al Post-mortem imaging as an alternative to autopsy in the diagnosis of adult deaths: a validation study. Lancet (London, England) 2012;379:136–42. 10.1016/S0140-6736(11)61483-9 22112684 PMC3262166

[11] Castillo P, Ussene E, Ismail MR, Jordao D, Lovane L, Carrilho C, et al Pathological Methods Applied to the Investigation of Causes of Death in Developing Countries: Minimally Invasive Autopsy Approach. PLoSOne 2015;10:e0132057. 10.1371/journal.pone.0132057 PMC4488344 26126191

[12] Martinez MJ, Massora S, Mandomando I, Ussene E, Jordao D. Infectious cause of death determination using minimally invasive autopsies in developing countries. Diagn Microbiol Infect Dis 2016;84:80–6. 10.1016/j.diagmicrobio.2015.10.002 26508103

[13] Castillo P, Martinez MJ, Ussene E, Jordao D, Lovane L, Ismail MR, et al Validity of a Minimally Invasive Autopsy for Cause of Death Determination in Adults in Mozambique: An Observational Study. PLOS Med 2016;13:e1002171 10.1371/journal.pmed.1002171 27875530 PMC5119723

[14] Bassat Q, Castillo P, Martínez MJ, Jordao D, Lovane L, Hurtado JC, et al Validity of a minimally invasive autopsy tool for cause of death determination in pediatric deaths in Mozambique: An observational study. PLOS Med 2017;14:e1002317 10.1371/journal.pmed.1002317 28632739 PMC5478091

[15] Hutchins GM, Berman JJ, Moore GW, Hanzlick R. Practice guidelines for autopsy pathology: autopsy reporting. Autopsy Committee of the College of American Pathologists. ArchPatholLab Med 1999;123:1085–92. 10.5858/1999-123-1085-PGFAP 10539932

[16] Grinberg LT, Ferraz da Silva LF, Galtarossa Xavier AC, Nascimento Saldiva PH, Mauad T. Clinico-pathological discrepancies in the diagnoses of solid malignancies. Pathol Res Pract 2008;204:867–73. 10.1016/j.prp.2008.07.001 18755553

[17] Goldman L, Sayson R, Robbins S, Cohn LH, Bettmann M, Weisberg M. The value of the autopsy in three medical eras. NEnglJMed 1983;308:1000–5. 10.1056/NEJM198304283081704 6835306

[18] Battle RM, Pathak D, Humble CG, Key CR, Vanatta PR, Hill RB, et al Factors influencing discrepancies between premortem and postmortem diagnoses. JAMA 1987;258:339–44. 3599326

[19] Ordi J, Ismail MR, Carrilho C, Romagosa C, Osman N, Machungo F, et al Clinico-pathological discrepancies in the diagnosis of causes of maternal death in sub-Saharan Africa: retrospective analysis. PLoS Med 2009;6:e1000036 10.1371/journal.pmed.1000036 19243215 PMC2646780

[20] Landis JR, Koch GG. The measurement of observer agreement for categorical data. Biometrics 1977;33:159–74. 843571

[21] Firth D. Bias reduction of maximum likelihood estimates. Biometrika 1993;80:27–38. 10.1093/biomet/80.1.27

[22] Heinze G, Schemper M. A solution to the problem of separation in logistic regression. Stat Med 2002;21:2409–19. 10.1002/sim.1047 12210625

[23] Sarode VR, Datta BN, Banerjee AK, Banerjee CK, Joshi K, Bhusnurmath B, et al Autopsy findings and clinical diagnoses: a review of 1,000 cases. HumPathol 1993;24:194–8. 10.1016/0046-8177(93)90300-6 8432514

[24] Marshall HS, Milikowski C. Comparison of Clinical Diagnoses and Autopsy Findings: Six-Year Retrospective Study. Arch Pathol Lab Med 2017;141:1262–6. 10.5858/arpa.2016-0488-OA 28657772

[25] Pastores SM, Dulu A, Voigt L, Raoof N, Alicea M, Halpern NA. Premortem clinical diagnoses and postmortem autopsy findings: discrepancies in critically ill cancer patients. Crit Care 2007;11:R48 10.1186/cc5782 17448238 PMC2206477

[26] Sonderegger-Iseli K, Burger S, Muntwyler J, Salomon F. Diagnostic errors in three medical eras: a necropsy study. Lancet (London, England) 2000;355:2027–31. 10.1016/s0140-6736(00)02349-7 10885353

[27] Kassirer JP, Kopelman RI. Cognitive errors in diagnosis: instantiation, classification, and consequences. Am J Med 1989;86:433–41. 10.1016/0002-9343(89)90342-2 2648823

[28] Voytovich AE, Rippey RM, Suffredini A. Premature conclusions in diagnostic reasoning. J Med Educ 1985;60:302–7. 3981589 10.1097/00001888-198504000-00004

[29] Zimmermann-Hösli MB, Stahel RA, Vogt P, Oelz O. Reduction of systemic fungal infections in patients with hematological malignancies, neutropenia, and prolonged fever by early amphotericin B therapy. Klin Wochenschr 1988;66:1010–4. 10.1007/bf01733443 3236752

[30] Cox JA, Lukande RL, Nelson AM, Mayanja-Kizza H, Colebunders R, Van Marck E, et al An Autopsy Study Describing Causes of Death and Comparing Clinico-Pathological Findings among Hospitalized Patients in Kampala, Uganda. PLoS One 2012;7:e33685 10.1371/journal.pone.0033685 22432042 PMC3303855

[31] Cohen MC, Paley MN, Griffiths PD, Whitby EH. Less invasive autopsy: benefits and limitations of the use of magnetic resonance imaging in the perinatal postmortem. PediatrDevPathol 2008;11:1–9. 10.2350/07-01-0213.1 18237232

[32] Perkins MD, Cunningham J. Facing the crisis: improving the diagnosis of tuberculosis in the HIV era. J Infect Dis 2007;196 Suppl:S15–27. 10.1086/518656 17624822

[33] Cohen GM. Access to diagnostics in support of HIV/AIDS and tuberculosis treatment in developing countries. AIDS 2007;21 Suppl 4:S81–87. 10.1097/01.aids.0000279710.47298.5c 17620757

[34] Murray CJ, Lozano R, Flaxman AD, Serina P, Phillips D, Stewart A, et al Using verbal autopsy to measure causes of death: the comparative performance of existing methods. BMC Med 2014;12:5 10.1186/1741-7015-12-5 24405531 PMC3891983

[35] Anker M. The effect of misclassification error on reported cause-specific mortality fractions from verbal autopsy. Int J Epidemiol 1997;26:1090–6. 10.1093/ije/26.5.1090 9363532

[36] Chandramohan D, Setel P, Quigley M. Effect of misclassification of causes of death in verbal autopsy: can it be adjusted? Int J Epidemiol 2001;30:509–14. 10.1093/ije/30.3.509 11416073

[37] Ravakhah K. Death certificates are not reliable: revivification of the autopsy. South Med J 2006;99:728–33. 10.1097/01.smj.0000224337.77074.57 16866055

[38] Fottrell E, Byass P, Ouedraogo TW, Tamini C, Gbangou A, Sombié I, et al Revealing the burden of maternal mortality: a probabilistic model for determining pregnancy-related causes of death from verbal autopsies. Popul Health Metr 2007;5:1 10.1186/1478-7954-5-1 17288607 PMC1802065

