# Supplementary material for: Clinico-pathological discrepancies in the diagnosis of causes of death in adults in Mozambique: A retrospective observational study
Source: PLoS One. 2019 Sep 6;14(9):e0220657. doi: 10.1371/journal.pone.0220657 (PMC6730941; doi:10.1371/journal.pone.0220657)
Supplement: S1 Table — (DOCX) [file pone.0220657.s001.docx]

| **Case** | **Sex** | **Age** | **HIV (1)** | **Clinical diagnosis (1)** | **Clinical diagnosis (2)** | **Clinical diagnosis (3)** | **Clinical diagnosis (4)** | **Clinical diagnosis (5)** | **Autopsy main diagnosis** | **HIV (2)** | **Error** |
| --- | --- | --- | --- | --- | --- | --- | --- | --- | --- | --- | --- |
| 1 | M | 27 | + | Bacterial sepsis | AIDS | Diabetes |  |  | Mucormycosis | - | Class I |
| 2 | M | 76 | - | Stroke | Febrile syndrome |  |  |  | Cerebral infarction | - | Class V |
| 3 | M | 30 | ND | Liver cell carcinoma |  |  |  |  | Liver cell carcinoma | - | Class V |
| 4 | F | 28 | + | Kaposi sarcoma | Pulmonary tuberculosis | AIDS |  |  | Disseminated Kaposi sarcoma | + | Class V |
| 5 | F | 35 | + | Acute gastroenteritis | Pneumonia | AIDS | Anemia |  | Pneumocystosis | + | Class I |
| 6 | M | 49 | ND | Type 2 diabetes mellitus | Stroke |  |  |  | Diabetes mellitus ketoacidosis | - | Class V |
| 7 | F | 35 | + | Meningitis | Pneumonia | AIDS |  |  | Toxoplasmosis | + | Class I |
| 8 | F | 27 | + | AIDS | Kaposi Sarcoma | Dilated cardiomyopathy | Renal insufficiency | Hypertension | Bacterial pneumonia | + | Class I |
| 9 | M | 41 | ND | Tuberculosis meningitis | Bronchopneumonia |  |  |  | Pneumococcal meningitis | + | Class I |
| 10 | M | 33 | + | Meningitis | AIDS |  |  |  | Cytomegaloviral disease | + | Class I |
| 11 | F | 76 | ND | Stroke | Hypertension | Aspiration pneumonia |  |  | Intracerebral hemorrhage | - | Class V |
| 12 | F | 17 | + | Metabolic encephalopathy | Cerebral tumor | Traditional drug toxicity | Aspiration pneumonia | AIDS | Tuberculous meningitis | + | Class I |
| 13 | M | 43 | + | Encephalitis (viral) | Renal insufficiency | AIDS | Anemia (severe) |  | Pneumocystosis | + | Class I |
| 14 | M | 48 | ND | Liver cell carcinoma | Renal insufficiency |  |  |  | Liver cell carcinoma | - | Class V |
| 15 | F | 53 | + | Toxoplamosis | Renal insufficiency | AIDS |  |  | B-cell lymphoma | + | Class I |
| 16 | F | 36 | + | Chronic renal failure | Carcinoma of cervix uteri | AIDS | Anemia |  | Carcinoma of cervix uteri | + | Class V |
| 17 | M | 62 | ND | Hypertensive encephalopathy | Hypertension |  |  |  | Cerebral infarction | - | Class V |
| 18 | M | 35 | + | Pulmonary tuberculosis | Anemia (severe) | Acute psychosis | Digestive bleeding | AIDS | Adenoviral pneumonia | + | Class I |
| 19 | F | 29 | + | Acute gastroenteritis | AIDS | Renal insufficiency | Anemia |  | Miliary tuberculosis | + | Class I |
| 20 | F | 61 | ND | Hypertensive encephalopathy | Renal insufficiency |  |  |  | Tuberculous meningitis | - | Class I |
| 21 | M | 74 | ND | Gastrointestinal hemorrhage | Congestive heart failure |  |  |  | Gastric ulcer with hemorrhage | - | Class II |
| 22 | F | 57 | + | Anemia | Renal insufficiency | Carcinoma cervix uteri | AIDS |  | Carcinoma cervix uteri | + | Class V |
| 23 | F | 45 | + | Meningitis | Anemia (severe) |  |  |  | Miliary tuberculosis | + | Class I |
| 24 | F | 29 | + | Meningitis | AIDS |  |  |  | Disseminated cryptococcosis | + | Class I |
| 25 | M | 32 | + | Steven-Johnson syndrome | AIDS | Tuberculosis |  |  | Other streptococcal sepsis | + | Class I |
| 26 | F | 30 | + | Cryptococcal meningitis | AIDS | Diarrhea |  |  | Toxoplasma meningoencephalitis | + | Class I |
| 27 | M | 38 | ND | Pneumonia | Anemia (severe) |  |  |  | Pneumonia (K. pneumoniae) | + | Class V |
| 28 | F | 70 | - | Liver cell carcinoma | Renal insufficiency |  |  |  | Liver cell carcinoma | - | Class V |
| 29 | M | 73 | ND | Obstructive lung disease | Pneumonia | Renal insufficiency |  |  | Pneumoconiosis | - | Class I |
| 30 | M | 32 | + | Meningitis | Pulmonary tuberculosis | Anemia |  |  | Other sepsis | + | Class I |
| 31 | M | 39 | + | Abdominal mass | AIDS | Renal insufficiency |  |  | Diffuse large B-cell lymphoma | + | Class V |
| 32 | M | 37 | + | Pneumonia | Bacterial meningitis | AIDS |  |  | Bacterial sepsis | + | Class IV |
| 33 | F | 60 | ND | Pneumonia | Angor pectoris |  |  |  | Acute myocardial infarction | + | Class IV |
| 34 | F | 29 | + | Encephalitis | Pulmonary tuberculosis | AIDS |  |  | Pneumonia | + | Class I |
| 35 | F | 32 | ND | Stroke | Atrial fibrillation |  |  |  | Toxoplasmosis | + | Class I |
| 36 | F | 57 | ND | Coma uremia |  |  |  |  | Sepsis (Gram-negative bacteria) | + | Class I |

| **Case** | **Sex** | **Age** | **HIV (1)** | **Clinical diagnosis (1)** | **Clinical diagnosis (2)** | **Clinical diagnosis (3)** | **Clinical diagnosis (4)** | **Clinical diagnosis (5)** | **Autopsy main diagnosis** | **HIV (2)** | **Error** |
| --- | --- | --- | --- | --- | --- | --- | --- | --- | --- | --- | --- |
| 37 | M | 34 | ND | Meningitis |  |  |  |  | Disseminated cryptococcosis | + | Class I |
| 38 | F | 44 | + | Pneumonia | AIDS | Acute renal failure | Gastroenteritis | Toxoplasmosis | Pneumonia ( Escherichia coli) | + | Class V |
| 39 | M | 50 | + | Kaposi sarcoma | AIDS | Chronic renal failure | Pulmonary tuberculosis | Gastroenteritis | Miliary tuberculosis | + | Class V |
| 40 | M | 36 | + | Cryptococcal meningitis | Cytomegaloviral retinitis | AIDS | Renal insufficiency | Liver failure | Disseminated cryptococcosis | + | Class V |
| 41 | M | 36 | + | Anemia (severe) | Acute renal failure | AIDS |  |  | Pneumonia (K. pneumoniae) | + | Class I |
| 42 | M | 19 | ND | Meningitis |  |  |  |  | Bacterial meningitis | - | Class V |
| 43 | M | 50 | ND | Cerebral malaria | Respiratory insufficiency |  |  |  | Pneumococcal meningitis | + | Class I |
| 44 | F | 55 | + | Encephalitis | AIDS | Cerebral hygroma | Renal insufficiency |  | Disseminated herpesviral disease | + | Class V |
| 45 | F | 50 | ND | Pulmonary tuberculosis | Diabetes mellitus |  |  |  | Respiratory tuberculosis | - | Class V |
| 46 | M | 51 | - | Cerebral tumor | Brain abscess |  |  |  | Herpesviral meningitis | - | Class I |
| 47 | F | 40 | + | Chronic renal failure | Malaria | AIDS | Anemia |  | Cytomegaloviral pneumonitis | + | Class I |
| 48 | F | 33 | ND | Cerebral malaria | Gastroenteritis |  |  |  | Gastroenteritis | - | Class V |
| 49 | M | 34 | + | Anemia (severe) | AIDS | Hypoglycemic coma | Tuberculous meningitis |  | Sepsis (Gram-negative bacteria) | + | Class I |
| 50 | F | 32 | + | Kaposi sarcoma | AIDS | Anemia |  |  | Gastroenteritis | + | Class I |
| 51 | M | 28 | + | Anemia (severe) | Toxic hepatitis | AIDS | Renal insufficiency |  | Other sepsis | - | Class I |
| 52 | M | 15 | ND | Intestinal occlusion | Appendicitis |  |  |  | Candidal sepsis | + | Class I |
| 53 | M | 44 | ND | Hypertensive encephalopathy | Acute renal failure |  |  |  | Disseminated cryptococcosis | + | Class I |
| 54 | M | 42 | ND | Chronic renal failure | Anemia (severe) | Cardiac insufficiency |  |  | Thrombotic microangiopathy | - | Class I |
| 55 | F | 34 | + | Meningitis | Anemia (severe) | AIDS | Acute renal failure |  | Respiratory tuberculosis | + | Class I |
| 56 | M | 46 | + | Pneumonia | Anemia (severe) | AIDS | Acute renal failure | Hypoglycemia | Myeloid leukemia | + | Class I |
| 57 | F | 28 | - | Gynecological peritonitis | Sepsis | Anemia (severe) | Acute renal failure |  | Bacterial sepsis | - | Class V |
| 58 | F | 24 | ND | Anemia (severe) |  |  |  |  | Respiratory tuberculosis | - | Class I |
| 59 | M | 55 | + | Meningitis | AIDS |  |  |  | Salmonella sepsis | + | Class I |
| 60 | F | 37 | + | Congestive heart failure | AIDS | Chronic renal failure | Hypoglycemia |  | Dilated cardiomyopathy | + | Class IV |
| 61 | M | 17 | ND | Cerebral malaria | Hypoglycemia | Anuria |  |  | Pneumococcal meningitis | - | Class I |
| 62 | F | 25 | ND | Chronic renal failure | Hypertensive heart | Pneumonia |  |  | Hypertensive renal failure | - | Class V |
| 63 | M | 43 | + | Meningitis | AIDS |  |  |  | Disseminated cryptococcosis | + | Class I |
| 64 | M | 54 | + | Chronic diarrhea | AIDS | Pancytopenia | Pulmonary tuberculosis |  | Sepsis (Gram-negative bacteria) | + | Class I |
| 65 | M | 25 | ND | Malaria (severe) | Diarrhea |  |  |  | Disseminated cryptococcosis | + | Class I |
| 66 | F | 40 | + | Meningitis | AIDS |  |  |  | Toxoplasmosis, unspecified | + | Class I |
| 67 | M | 59 | + | Pneumocystis pneumonia | AIDS | Chronic renal failure | Anemia | Hypoglycemia | Pulmonary toxoplasmosis | + | Class I |
| 68 | F | 44 | ND | Cervix cancer | Renal insufficiency |  |  |  | Carcinoma of cervix uteri | - | Class V |
| 69 | F | 36 | + | Meningitis | Anemia (severe) | AIDS |  |  | Legionnaires disease | + | Class I |
| 70 | F | 35 | + | Cryptococcal meningitis | AIDS |  |  |  | Cerebral cryptococcosis | + | Class V |
| 71 | F | 36 | ND | Submandibular abscess | Lung mass |  |  |  | Acute pericarditis | - | Class I |
| 72 | F | 46 | + | Sepsis | AIDS | Pulmonary tuberculosis | Gangrene | Renal failure | Miliary tuberculosis | + | Class V |
| **Case** | **Sex** | **Age** | **HIV (1)** | **Clinical diagnosis (1)** | **Clinical diagnosis (2)** | **Clinical diagnosis (3)** | **Clinical diagnosis (4)** | **Clinical diagnosis (5)** | **Autopsy main diagnosis** | **HIV (2)** | **Error** |
| 73 | M | 21 | - | Rabies |  |  |  |  | Rabies | - | Class V |
| 74 | F | 45 | ND | Stroke | Pneumonia | Diabetes mellitus |  |  | Intracerebral hemorrhage | + | Class IV |
| 75 | M | 40 | ND | Meningitis | AIDS | Tuberculosis |  |  | Sepsis (Gram-negative bacteria) | - | Class I |
| 76 | F | 32 | + | Cryptococcal meningitis | AIDS | Pneumocystis | Anemia | Chronic diarrhea | Miliary tuberculosis | + | Class I |
| 77 | M | 16 | ND | Meningitis |  |  |  |  | Other sepsis | - | Class III |
| 78 | F | 23 | + | Meningitis | AIDS |  |  |  | Toxoplasma meningoencephalitis | + | Class I |
| 79 | F | 52 | - | Diabetic ketoacidosis | Acute renal failure |  |  |  | Diabetes mellitus. Ketoacidosis | - | Class V |
| 80 | F | 48 | - | Stroke | Pneumonia | Syphilis | Traditional drug toxicity | Hypertension | Pneumonia (Mycoplasma pneumoniae) | - | Class V |
| 81 | F | 58 | + | Dilated cardiomyopathy | Abdominal mass | AIDS |  |  | Malignant neoplasm, site not specified | + | Class I |
| 82 | F | 33 | + | Hepatic encephalopathy | AIDS | Tuberculosis | Renal insufficiency | Cholestasis | Miliary tuberculosis | + | Class V |
| 83 | M | 34 | + | Pneumonia | AIDS | Tuberculosis |  |  | Disseminated Kaposi sarcoma | + | Class I |
| 84 | F | 23 | + | Kaposi sarcoma | Chronic diarrhea | Anemia |  |  | Pneumonia (K. pneumoniae) | - | Class I |
| 85 | M | 40 | + | Hemorrhagic stroke | Hypertension | AIDS | Aspiration pneumonia |  | Pneumonia (Escherichia coli) | + | Class V |
| 86 | F | 56 | + | Cerebral lesion | Pulmonary tuberculosis | AIDS |  |  | Intracerebral eaemorrhage | + | Class II |
| 87 | M | 31 | ND | Pericardial effusion | Pericardial tamponade |  |  |  | Infective pericarditis | - | Class V |
| 88 | F | 39 | + | Abdominal mass | Ascites | AIDS |  |  | Diffuse large B-cell lymphoma | + | Class V |
| 89 | M | 35 | + | Cerebral lesion | AIDS |  |  |  | Bacterial sepsis | - | Class I |
| 90 | F | 30 | + | Abdominal mass | AIDS | Acute renal failure | Anemia |  | Liver cell carcinoma | + | Class IV |
| 91 | F | 18 | - | Meningitis | Retroauricular abscess |  |  |  | Respiratory tuberculosis | - | Class I |
| 92 | M | 38 | + | Tuberculosis meningitis | AIDS |  |  |  | Miliary tuberculosis | + | Class V |
| 93 | M | 46 | + | Chronic renal failure | Gastroenteritis | AIDS | Anemia |  | Miliary tuberculosis | + | Class I |
| 94 | F | 16 | - | Chronic renal failure | Heart failure | Anemia |  |  | Pneumonia (Gram- bacteria) | - | Class I |
| 95 | M | 26 | - | Gastrointestinal bleeding | Anemia | Cirrhosis | Hypoglycemia |  | Esophageal varices with bleeding | - | Class V |
| 96 | M | 52 | + | Coma hypoglycemia | Acute renal failure | AIDS | Anemia | Bronchopneumonia | Pneumonia | + | Class V |
| 97 | F | 46 | + | Pneumocystis pneumonia | Abdominal mass | Acute renal failure |  |  | Miliary tuberculosis | + | Class I |
| 98 | M | 38 | + | Pneumonia | Pulmonary tuberculosis | AIDS | Pancytopenia |  | Miliary tuberculosis | + | Class V |
| 99 | M | 33 | + | Abdominal mass | AIDS | Anemia | Acute renal failure |  | Miliary tuberculosis | + | Class I |
| 100 | M | 49 | + | Bronchopneumonia | AIDS |  |  |  | Pneumonia (Pseudomonas) | + | Class V |
| 101 | F | 58 | ND | Stroke | Hypertension |  |  |  | Malignant neoplasm of brain | - | Class I |
| 102 | F | 29 | + | Meningitis | AIDS | Vulvar cancer |  |  | Toxoplasmosis | + | Class I |
| 103 | F | 38 | + | Cryptococcal meningitis | Pneumonia | AIDS |  |  | Toxoplasmosis | + | Class I |
| 104 | F | 28 | - | Asthma crisis |  |  |  |  | Interstitial pulmonary disease | - | Class V |
| 105 | M | 39 | ND | Liver cell carcinoma | Anemia (severe) | Hypoglycemia |  |  | Liver cell carcinoma | - | Class V |
| 106 | M | 49 | + | Cardiac arrest | AIDS |  |  |  | Cardiac arrest | - | Class V |
| 107 | M | 35 | + | Pericarditis | AIDS |  |  |  | Miliary tuberculosis | + | Class I |
| 108 | M | 47 | + | Meningitis | Pneumonia | Renal insufficiency | Anemia | AIDS | Miliary tuberculosis | + | Class I |
| **Case** | **Sex** | **Age** | **HIV (1)** | **Clinical diagnosis (1)** | **Clinical diagnosis (2)** | **Clinical diagnosis (3)** | **Clinical diagnosis (4)** | **Clinical diagnosis (5)** | **Autopsy main diagnosis** | **HIV (2)** | **Error** |
| 109 | M | 32 | + | Pneumonia | Pancytopenia | AIDS | Renal insufficiency |  | Miliary tuberculosis | + | Class I |
| 110 | M | 26 | - | Bronchopneumonia |  |  |  |  | Respiratory tuberculosis | - | Class I |
| 111 | M | 36 | + | Pulmonary tuberculosis | Anemia (severe) | AIDS |  |  | Miliary tuberculosis | + | Class V |
| 112 | M | 26 | + | Pulmonary tuberculosis | Anemia | AIDS |  |  | Miliary tuberculosis | + | Class V |

Age, sex, HIV status in the clinical records, clinical diagnoses (up to five) registered in the clinical records, autopsy diagnosis, HIV status at autopsy and final assessment of the clinico-pathological discrepancy of each particular patient.

HIV (1): HIV antibodies detected clinically; HIV (2): HIV antibodies detected at autopsy; +: positive; -: negative; ND: not done.
