# Supplementary material for: Clinico-pathological discrepancies in the diagnosis of causes of death in adults in Mozambique: A retrospective observational study
Source: PLoS One. 2019 Sep 6;14(9):e0220657. doi: 10.1371/journal.pone.0220657 (PMC6730941; doi:10.1371/journal.pone.0220657)
Supplement: S1 Form — (PDF) [file pone.0220657.s002.pdf]

cism centro de investigação em saúde de manhiça	***Hospital Central de Maputo (HCM)*** ***Centro de Investigação em Saúde da Manhiça (CISM)*** **RECOLHA DADOS CLÍNICOS** **CaDMIA-POSTMORTEM** SSP_CAD_HO_001_A03_v01_PT	ECADM	***Número de serie***

Número de estudo: CADM-|__|__|__|__|

Número de estudo
Colar etiqueta aquí

Informação pessoal

1. Hora (24h) e data (dd/mm/aaaa) do internamento |__|__|.|__|__| dia |__|__|/|__|__|/|__|__|__|__|
2. Hora (24h) e data (dd/mm/aaaa) da morte |__|__|.|__|__| dia |__|__|/|__|__|/|__|__|__|__|
3. Lugar de recrutamento Maputo (Moçambique) ☐ Manaus (Brasil) ☐
4. Número do prontuário do ambulatório/Processo clínico |__|__| / |__|__|__|__|__|__| ND ☐
5. Nome do paciente |__|__|__|__|__|__|__|__|__|__|__|__|__|__|__|__|__|__|__|__|__|__|__|__|__|
6. Sexo Homem ☐ Mulher ☐
7. Procedência do paciente Meio Urbano ☐ Rural ☐ ND ☐
8. Grupo étnico Branco ☐ Mestiço ☐ Negro ☐ Indígena ☐ Amarelo ☐
9. Em qué grupo do estudo estaría o doente recrutado?
   Mortinatos ☐ Neonatos ☐ Crianças<12 anos ☐ Mulheres em idade fértil ☐ Outros adultos ☐
10. Status do paciente em termos de gravidez?
    Não grávida ☐ Grávida ☐ Aborto ☐ Parto ☐ Puérpera ☐ Gravidez Ectópica ☐ N/A ☐
11. Data de nascimento do paciente
    (dd/mm/aaaa) |__|__|/|__|__|/|__|__|__|__| ND ☐ **OU** Idade (anos) |__|__| ND ☐
12. Assinou a família o Consentimento informado (IC ou TCLE)? Não ☐ Sim ☐ N/A ☐
13. Numero de autopsia (HCM) **A**-|__|__| / |__|__|__|__|

Anamnese na chegada ao hospital (preencher para todos excepto mortinatos) (ND=Não Disponível)

14. Febre referida Não ☐ Sim ☐ ND ☐ N° de dias |__|__| ND ☐
15. Tosse Não ☐ Sim ☐ ND ☐ N° de dias |__|__| ND ☐
16. Dispneia Não ☐ Sim ☐ ND ☐ N° de dias |__|__| ND ☐
17. Diarreia Não ☐ Sim ☐ ND ☐ N° de dias |__|__| ND ☐
18. *Se sim, características das fezes Aquosas ☐ Com sangue/muco ☐ ND ☐
19. Vómitos Não ☐ Sim ☐ ND ☐ N° de dias |__|__| ND ☐
20. Convulsões Não ☐ Sim ☐ ND ☐ N.º episódios últimas 24h |__|__| ND ☐
21. Cefaleia Não ☐ Sim ☐ ND ☐ N° de dias |__|__| ND ☐
22. Calafrios Não ☐ Sim ☐ ND ☐ N° de dias |__|__| ND ☐
23. Dor abdominal Não ☐ Sim ☐ ND ☐ N° de dias |__|__| ND ☐
24. Oliguria/ Anuria Não ☐ Sim ☐ ND ☐ N° de dias |__|__| ND ☐
25. Coluria Não ☐ Sim ☐ ND ☐ N° de dias |__|__| ND ☐

26. Sangue na orina Não ☐ Sim ☐ ND ☐ N° de dias |__|__| ND ☐
27. Sangramento vaginal Não ☐ Sim ☐ ND ☐ N/A ☐ N° de dias |__|__| ND ☐
28. Corrimento vaginal Não ☐ Sim ☐ ND ☐ N/A ☐ N° de dias |__|__| ND ☐
29. Amenorrea Não ☐ Sim ☐ ND ☐ N/A ☐ N° de meses |__|__| ND ☐
30. Outros Sintomas? Não ☐ Sim ☐ ND ☐
31. *(Especificar 1) |__|__|__|__|__|__|__|__|__|__|__|__|__|__|__|__|__|__|__|__|__|__|__|__|
32. *(Especificar 2) |__|__|__|__|__|__|__|__|__|__|__|__|__|__|__|__|__|__|__|__|__|__|__|__|
33. Tem antecedente de alcoolismo? Não ☐ Sim ☐ ND ☐
34. Tem antecedente como fumador? Não ☐ Sim ☐ ND ☐
35. Tem outros antecedentes destacáveis? Não ☐ Sim ☐
36. *(Especificar1) |__|__|__|__|__|__|__|__|__|__|__|__|__|__|__|__|__|__|__|__|__|__|__|__|
37. *(Especificar2) |__|__|__|__|__|__|__|__|__|__|__|__|__|__|__|__|__|__|__|__|__|__|__|__|
38. Tem doenças crónicas conhecidas? Não ☐ Sim ☐ ND ☐
39. Diabetes Não ☐ Sim ☐ ND ☐
40. Hipertensão arterial Não ☐ Sim ☐ ND ☐
41. Cardiopatia Não ☐ Sim ☐ ND ☐
42. Insuficiência renal crónica Não ☐ Sim ☐ ND ☐
43. HIV/SIDA Não ☐ Sim ☐ ND ☐
44. Tuberculose Não ☐ Sim ☐ ND ☐
45. Outras Não ☐ Sim ☐
46. *(Especificar 1) |__|__|__|__|__|__|__|__|__|__|__|__|__|__|__|__|__|__|__|__|__|__|__|__|
47. *(Especificar 2) |__|__|__|__|__|__|__|__|__|__|__|__|__|__|__|__|__|__|__|__|__|__|__|__|
48. Toma de medicamentos pre-admissão? Não ☐ Sim ☐ ND ☐
49. Antibióticos Não ☐ Sim ☐ ND ☐
50. *(Especificar 1) |__|__|__|__|__|__|__|__|__|__|__|__|__|__|__|__|__|__|__|__|__|__|__|__|
51. *(Especificar 2) |__|__|__|__|__|__|__|__|__|__|__|__|__|__|__|__|__|__|__|__|__|__|__|__|
52. Tratamento antituberculose Não ☐ Sim ☐ ND ☐
53. Antirretrovirais Não ☐ Sim ☐ ND ☐
54. *Se sim, quantos meses? |__|__|__| ND ☐
55. Antimaláricos Não ☐ Sim ☐ ND ☐
56. Diuréticos/Antihipertensivos Não ☐ Sim ☐ ND ☐
57. Insulina/Tratamento diabetes Não ☐ Sim ☐ ND ☐
58. Anticonvulsivantes Não ☐ Sim ☐ ND ☐
59. Outros Não ☐ Sim ☐
60. *(Especificar) |__|__|__|__|__|__|__|__|__|__|__|__|__|__|__|__|__|__|__|__|__|__|__|__|

Sinais clínicos na chegada ao Hospital (preencher para todos excepto mortinatos)

61. Peso (kg) |__|__|__|.|__| ND ☐
62. Cumprimento (cms) |__|__|__| ND ☐
63. Estado nutricional Normal ☐ Caquexia/Malnutrição ☐ Obesidade ☐ ND ☐ N/A ☐

64. Temperatura Axilar (ºC) |__|__| . |__| ND ☐
65. Frequência respiratória (avaliar num minuto) |__|__|__| ND ☐
66. Frequência cardíaca (avaliar num minuto) |__|__|__| ND ☐
67. Pressão arterial (sistólica/diástolica) |__|__|__|/|__|__|__| ND ☐
68. Desidratação Não ☐ Leve ☐ Moderada ☐ Grave ☐ ND ☐
69. Edemas Não ☐ Sim ☐ ND ☐
70. Adenopatias Não ☐ Sim ☐ ND ☐
71. Palidez Não ☐ Sim ☐ ND ☐
72. Candidíase oral Não ☐ Sim ☐ ND ☐
73. Icterícia Não ☐ Sim ☐ ND ☐

74. Fígado palpável Não ☐ Sim ☐ ND ☐ |__|__| cms ND ☐
75. Baço palpável Não ☐ Sim ☐ ND ☐ |__|__| cms ND ☐
76. Petequias, púrpura ou hematomas Não ☐ Sim ☐ ND ☐
77. Exantemas cutâneos ou outras lesões cutáneas Não ☐ Sim ☐ ND ☐

Exame neurológico na chegada ao Hospital (preencher para todos excepto mortinatos)

78. Estado geral Consciente ☐ Letárgia ☐ Confusão/Agitação ☐ Inconsciente ☐ ND ☐
79. Escala de COMA de Glasgow (se Adulto) |__|__| / 15 ND ☐ N/A ☐
80. Escala de COMA de Blantyre (se criança<12 anos) |__| / 5 ND ☐ N/A ☐
81. Rigidez de nuca/Síndrome meníngeo/Fontanela abaulada Não ☐ Sim ☐ ND ☐
82. Focalidade neurológica Não ☐ Sim ☐ ND ☐
83. *(Especificar) |__|__|__|__|__|__|__|__|__|__|__|__|__|__|__|__|__|__|__|__|__|__|__|__|__|
84. Evidencia de convulsões durante o internamento? Não ☐ Sim ☐ ND ☐

História obstétrica (Preencher só em caso de MORTE MATERNA OU PERINATAL)

85. Trata-se de um caso de morte materna ou perinatal? Não ☐ Sim ☐
(se Sim, seguir preenchendo, se não passar ao seguinte bloco)
86. Número de gestações (incluindo a gravidez atual) |__|__| ND ☐
87. TPAL* (Se primeira gestação, TPAL= 0-0-0-0) (T=Partos a término ; P=Partos prematuros ; A=Abortos, L=Rescem nascidos vivos) **T|__|-P|__|-A|__|-L|__|** ND ☐
88. **G-P-A-Fv-Ff**: Gestaçoes |__|__| Paridade |__|__| Abortos |__| Filhos vivos |__|__| Filhos falecidos |__| ND ☐
89. Antecedente de cesariana prévia? Não ☐ Sim ☐ ND ☐
90. Idade gestacional (no momento do aborto/parto/morte) |__|__| Semanas e |__| dias ND ☐
91. Quando aconteceu a morte? Antes do parto ☐ Durante o parto/aborto ☐ Após o parto/aborto ☐
92. Febre materna durante a gravidez Não ☐ Sim ☐ ND ☐
93. Malária materna durante a gravidez Não ☐ Sim ☐ ND ☐
94. Despistagem Sifilis (RPR ou VRDL) durante a gravidez Negativo ☐ Positivo ☐ ND ☐
95. Anemia materna (Hb<7g/dL) durante gravidez, parto ou puerperio Não ☐ Sim ☐ ND ☐
96. Outros dados obstétricos Não ☐ Sim ☐
Eclampsia/Preeclampsia ☐ Ameaça aborto ☐ Diabetes gestacional ☐ Outros ☐
Se outros, especificar |__|__|__|__|__|__|__|__|__|__|__|__|__|__|__|__|__|__|__|__|__|__|__|__|

Parto (Preencher só em caso de MORTE MATERNA OU PERINATAL)

97. Trata-se de um caso de morte materna ou perinatal? Não ☐ Sim ☐
(se Sim, seguir preenchendo, se não passar ao seguinte bloco)

98. Resultado do parto? Nado vivo ☐ Nado morto (stillbirth) ☐ Aborto ☐ ND ☐ N/A ☐

99. Lugar do parto Casa ☐ Posto Saúde ☐ Hospital ☐ Outros ☐ ND ☐ N/A ☐

100. Pessoal a atender parto Parteira tradicional ☐ Enfermeira ☐ Médico ☐ Outros ☐ ND ☐ N/A ☐

101. Parto arrastrado? Sim ☐ Não ☐ ND ☐ N/A ☐

102. Apresentação fetal: Cefálica ☐ Podálica ☐ Transversa ☐ Gravidez múltiple ☐ ND ☐ N/A ☐

103. Tipo de parto Vaginal ☐ Cesariana ☐ N/A ☐

104. Febre intraparto? Não ☐ Sim ☐ ND ☐ N/A ☐

105. A mãe refere rotura das águas <9 meses gravidez? (rotura prematura) Não ☐ Sim ☐ ND ☐ N/A ☐

106. A mãe refere rotura das águas >24h? (rotura prolongada) Não ☐ Sim ☐ ND ☐ N/A ☐

107. Suspeita de águas infectadas (águas com cheiro o com pus)? Não ☐ Sim ☐ ND ☐ N/A ☐

108. A mãe recebeu tratamento antibiótico intraparto? Não ☐ Sim ☐ ND ☐ N/A ☐

109. Existe evidência de mecónio nas águas? Não ☐ Sim ☐ ND ☐ N/A ☐

História do recém nascido (só em caso de NADO MORTO ou NEONATO MORTO)

110. Trata-se de um caso de Nado morto ou Neonato morto? Não ☐ Sim ☐
(se Sim, seguir preenchendo, se não passar ao seguinte bloco)

111. Qué tipo de caso é? Nado morto macerado ☐ Nado morto recente ☐ Neonato morto ☐

112. Idade da mãe (anos) |__|__| ND ☐

113. Status HIV materno Negativo ☐ Positivo ☐ ND ☐

114. * Em caso de mãe HIV+, contagem mais recente de CD4s |__|__|__|__|__| ND ☐

115. * Em caso de mãe HIV+, está fazer TARV? Não ☐ Sim ☐ ND ☐

116. Peso ao nascer (em gramas) |__|__|__|__| gramas ND ☐

117. APGAR da criança ao nascer (minuto 1 e 5) |__|__| / |__|__| ND ☐ N/A ☐

118. Vitalidade da criança ao nascer

Normal (activa espontáneamente) ☐ Reactiva só e estimulada ☐ Ausente ☐ ND ☐ N/A ☐

119. A criança respirou espontaneamente? Não ☐ Sim ☐ ND ☐ N/A ☐

120. *Se não, foi necessária reanimação básica (Ambú, oxigênio)? Não ☐ Sim ☐ ND ☐ N/A ☐

121. A criança chorou logo ao nascer Não ☐ Sim ☐ ND ☐ N/A ☐

122. Apresenta alguma malformação congénita visível? Não ☐ Sim ☐ ND ☐

123. Se sim, descrever

1. |__|__|__|__|__|__|__|__|__|__|__|__|__|__|__|__|__|__|__|__|__|__|__|__|__|__|__|__|__|__|__|__|
2. |__|__|__|__|__|__|__|__|__|__|__|__|__|__|__|__|__|__|__|__|__|__|__|__|__|__|__|__|__|__|__|__|

124. Outros achados? 1. |__|__|__|__|__|__|__|__|__|__|__|__|__|__|__|__|__|__|__|__|__|__|__|__|__|__|
2. |__|__|__|__|__|__|__|__|__|__|__|__|__|__|__|__|__|__|__|__|__|__|__|__|__|__|
3. |__|__|__|__|__|__|__|__|__|__|__|__|__|__|__|__|__|__|__|__|__|__|__|__|__|__|

125. A criança presentó Apneias respiratórias? Não ☐ Sim ☐ ND ☐ N/A ☐

126. A criança buscava o mamilo da mãe? Não ☐ Sim ☐ ND ☐ N/A ☐

127. Ictericia Neonatal? Não ☐ Sim ☐ ND ☐ N/A ☐

128. Dificuldades respiratórias? Não ☐ Sim ☐ ND ☐ N/A ☐
129. Gemidos Não ☐ Sim ☐ ND ☐ N/A ☐
130. Onfalite Não ☐ Sim ☐ ND ☐ N/A ☐
131. Fontanela Normal ☐ Deprimida ☐ Abombada ☐ ND ☐ N/A ☐
132. Convulsões ou outros movimentos anómalos Não ☐ Sim ☐ ND ☐ N/A ☐
133. Tono muscular Normal ☐ Fraco (hipotonia) ☐ Exagerado (hipertonia) ☐ ND ☐ N/A ☐
134. Conjuntivite purulenta Não ☐ Sim ☐ ND ☐ N/A ☐

Evolução clínica durante admissão (Preencher para todos excepto para mortinatos)

135. Duração do internamento até morte (em dias) |__|__| dias
136. Febre durante a admissão? Não ☐ Sim ☐ ND ☐
137. Parasitémia Negat. ☐ + ☐ ++ ☐ +++ ☐ ++++ ☐ +++++ ☐ ND ☐
138. Teste rápido malária Negat. ☐ Positivo ☐ Indeterminado ☐ ND ☐
139. Status HIV Negativo ☐ Positivo ☐ Indeterminado ☐ ND ☐
140. Último contagem de CD4 (mais recente) |__|__|__|__|__| ND ☐
141. Hematócrito mais baixo detectado durante internamento |__|__|.|__| % ND ☐
142. Hemoglobina mais baixa detectada durante internamento |__|__|.|__| g/dL ND ☐
143. Recebeu transfusão/ões sanguíneas? Não ☐ Sim ☐ ND ☐
144. Contagem leucócitos mais alto durante internamento |__|__|. |__|__| 10^3/Ul ND ☐
145. Glicemia mais baixa detectada durante internamento |__|__|.|__| mmol/L ND ☐
146. Foi feita uma punção lumbar? Não ☐ Sim ☐ ND ☐
147. *Se sim, resultado Normal ☐ Patológico ☐ Punção traumática ☐ ND ☐
148. Foi feita uma fita de orina? Não ☐ Sim ☐ ND ☐
149. *Se sim, resultado
Normal ☐ Proteinas ☐ Glicosuria ☐ Bact/Piuria ☐ Leucos ☐ Levaduras ☐ Hematuria ☐ ND ☐
150. Foi feita hemocultura? Não ☐ Sim ☐ ND ☐
151. Foi feito RX Tórax? Não ☐ Sim ☐ ND ☐
152. *Se sim, resultado
Normal ☐ Patológico pulmões ☐ Patológico Coração ☐ Patológico outros ☐ ND ☐
153. *Se patológico, especificar:
|__|__|__|__|__|__|__|__|__|__|__|__|__|__|__|__|__|__|__|__|__|__|__|__|__|__|__|__|__|__|__|__|
154. Perfil hepático Normal ☐ Transaminasas altas ☐ Cholestase ☐ ND ☐
155. Perfil renal Normal ☐ Insuficiência renal ☐ ND ☐
156. BK despistagem tuberculose Negativo ☐ Positivo ☐ ND ☐
157. Outros achados laboratoriais importantes Não ☐ Sim ☐
158. *Se sim, especificar (1) |__|__|__|__|__|__|__|__|__|__|__|__|__|__|__|__|__|__|__|__|__|__|
159. *Se sim, especificar (2) |__|__|__|__|__|__|__|__|__|__|__|__|__|__|__|__|__|__|__|__|__|__|
160. *Se sim, especificar (3) |__|__|__|__|__|__|__|__|__|__|__|__|__|__|__|__|__|__|__|__|__|__|

Tratamentos recebidos durante a admissão
(Preencher para todos excepto para mortinatos)

161. Antimaláricos Não ☐ Sim ☐ ND ☐
162. Antibióticos Não ☐ Sim ☐ ND ☐
163. Penicilina Não ☐ Sim ☐ ND ☐
164. Cotrimoxazol Não ☐ Sim ☐ ND ☐
165. Cloramfenicol Não ☐ Sim ☐ ND ☐
166. Aminoglucosidos (Ex: Gentamicina) Não ☐ Sim ☐ ND ☐
167. Ampicilina/Amoxicilina Não ☐ Sim ☐ ND ☐
168. Macrolidos (ex: Eritromicina) Não ☐ Sim ☐ ND ☐
169. Cefalosporinas (Ex: Ceftriaxona) Não ☐ Sim ☐ ND ☐
170. Quinolonas (Ex: Ciprofloxacino) Não ☐ Sim ☐ ND ☐
171. Metronidazol Não ☐ Sim ☐ ND ☐
172. Outros Não ☐ Sim ☐
173. *Se outros, especificar (1): |__|__|__|__|__|__|__|__|__|__|__|__|__|__|__|__|__|__|__|__|
174. *Se outros, especificar (2): |__|__|__|__|__|__|__|__|__|__|__|__|__|__|__|__|__|__|__|__|
175. Tratamento Eclampsia Não ☐ Sim ☐ N/A ☐
176. Sulfato de Magnesio Não ☐ Sim ☐ ND ☐
177. Captopril Não ☐ Sim ☐ ND ☐
178. Nifedipino Não ☐ Sim ☐ ND ☐
179. Thiazidas Não ☐ Sim ☐ ND ☐
180. Methyldopa Não ☐ Sim ☐ ND ☐
181. Tratamento Antiretroviral (TARV) Não ☐ Sim ☐ ND ☐
182. Tratamento antituberculoso Não ☐ Sim ☐ ND ☐
183. Precisó Oxigenio? Não ☐ Sim ☐ ND ☐

Diagnósticos finais definidos por os clínicos (com códigos ICD10)

184. Diagnóstico Principal (e código) |__|__|__|__|__|__|__|__|__|__|__|__|__|__|__|__| |__|__|__|.|__|
185. Diagnóstico 2 (e código) |__|__|__|__|__|__|__|__|__|__|__|__|__|__|__|__|__| |__|__|__|.|__|
186. Diagnóstico 3 (e código) |__|__|__|__|__|__|__|__|__|__|__|__|__|__|__|__|__| |__|__|__|.|__|
187. Diagnóstico 4 (e código) |__|__|__|__|__|__|__|__|__|__|__|__|__|__|__|__|__| |__|__|__|.|__|
188. Diagnóstico 5 (e código) |__|__|__|__|__|__|__|__|__|__|__|__|__|__|__|__|__| |__|__|__|.|__|

Assinatura

189. Nome, assinatura e data (dd/mm/aaaa)

|__|__|__|__|__|__|__|__|__|__|__|__| ________________________ |__|__| / |__|__|/|__|__|__|__|
